# Structure, dynamics and immunogenicity of a catalytically inactive C*X*C chemokine-degrading protease SpyCEP from *Streptococcus pyogenes*

**DOI:** 10.1016/j.csbj.2020.03.004

**Published:** 2020-03-13

**Authors:** Sophie McKenna, Enrico Malito, Sarah L. Rouse, Francesca Abate, Giuliano Bensi, Emiliano Chiarot, Francesca Micoli, Francesca Mancini, Danilo Gomes Moriel, Guido Grandi, Danuta Mossakowska, Max Pearson, Yingqi Xu, James Pease, Shiranee Sriskandan, Immaculada Margarit, Matthew J. Bottomley, Stephen Matthews

**Affiliations:** a Department of Life Sciences, Imperial College London, South Kensington Campus, SW7 2AZ, UK; b GlaxoSmithKline, 14200 Shady Grove Road, Rockville, MD 20850, United States; c GSK Vaccines Institute for Global Health, Via Fiorentina 1, 53100 Siena, Italy; d Malopolska Centre of Biotechnology (MCB), Jagiellonian University Krakow, Gronostajowa 7a Str, 30-387 Krakow, Poland; e Department of Infectious Disease, Imperial College London, London W12 0NN, UK; f National Heart and Lung Institute, Imperial College London, London SW7 2AZ, UK; g GlaxoSmithKline, Via Fiorentina 1, 53100 Siena, Italy; h Department of Cellular, Computational and Integrative Biology (CIBIO), University of Trento, 38123 Trento, Italy; i Fast Trak Department, GE Healthcare, 75323 Uppsala, Sweden

## Abstract

Over 18 million disease cases and half a million deaths worldwide are estimated to be caused annually by Group A Streptococcus. A vaccine to prevent GAS disease is urgently needed. SpyCEP (Streptococcus *pyogenes* Cell-Envelope Proteinase) is a surface-exposed serine protease that inactivates chemokines, impairing neutrophil recruitment and bacterial clearance, and has shown promising immunogenicity in preclinical models. Although SpyCEP structure has been partially characterized, a more complete and higher resolution understanding of its antigenic features would be desirable prior to large scale manufacturing. To address these gaps and facilitate development of this globally important vaccine, we performed immunogenicity studies with a safety-engineered SpyCEP mutant, and comprehensively characterized its structure by combining X-ray crystallography, NMR spectroscopy and molecular dynamics simulations. We found that the catalytically-inactive SpyCEP antigen conferred protection similar to wild-type SpyCEP in a mouse infection model. Further, a new higher-resolution crystal structure of the inactive SpyCEP mutant provided new insights into this large chemokine protease comprising nine domains derived from two non-covalently linked fragments. NMR spectroscopy and molecular simulation analyses revealed conformational flexibility that is likely important for optimal substrate recognition and overall function. These combined immunogenicity and structural data demonstrate that the full-length SpyCEP inactive mutant is a strong candidate human vaccine antigen. These findings show how a multi-disciplinary study was used to overcome obstacles in the development of a GAS vaccine, an approach applicable to other future vaccine programs. Moreover, the information provided may also facilitate the structure-based discovery of small-molecule therapeutics targeting SpyCEP protease inhibition.

## Graphical abstract

**Figure:**
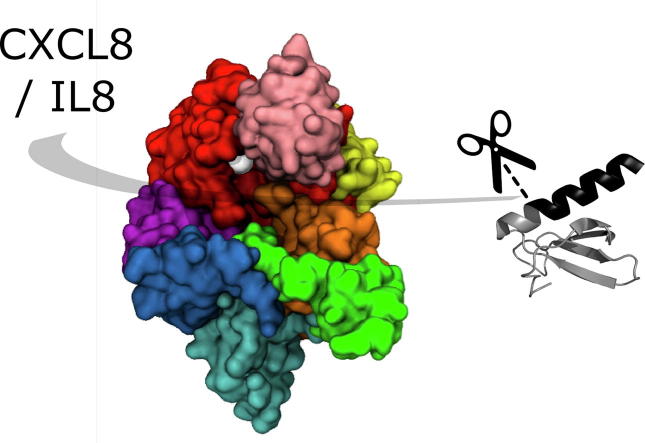

## 1 Introduction

*Streptococcus pyogenes* (Group A streptococcus; GAS) is an important human pathogen, responsible for a significant and diverse global disease burden from mild conditions such as impetigo and pharyngitis, through to life-threatening conditions such as acute rheumatic fever, rheumatic heart disease, streptococcal toxic shock syndrome and necrotising fasciitis [1]. The global disease burden attributed to *S. pyogenes* infection is estimated to be over 600 million cases annually, of which 18 million cases can be attributed to severe *S. pyogenes* infection, with approximately 1.8 million new cases, and over 517,000 deaths reported annually [2], [3].

*S. pyogenes* expresses an array of virulence factors to evade or inactivate the innate immune response [4]. Neutrophil recruitment and activation, mediated by the ELR+ group of chemokines and vital for bacterial clearance, is impaired in lethal soft-tissue *S. pyogenes* infections [5], [6]. The *S. pyogenes* Cell Envelope Proteinase (SpyCEP) is a highly conserved surface-exposed serine protease that cleaves and inactivates all ELR+ chemokines (containing the N-terminal glutamate leucine arginine motif), notably CXCL8 [7], [8], mediating evasion of immune system clearance strategies and systemic dissemination [9]. High level expression of SpyCEP correlates with disease severity and is indicative of a vital role in invasive *S. pyogenes* infection [8], [10].

SpyCEP is a 180 kDa heterodimer made of two non-covalently linked polypeptides derived from the full-length protein by an intramolecular autocatalytic processing event [11]. The N- and C-terminal fragments are 30 and 150 kDa respectively, and both contribute residues to the catalytic triad (D151 from the N-terminal fragment, H279 and S617 from the C-terminal fragment). The two components of SpyCEP can also be produced separately and reconstituted to generate the active enzyme [11], [12]. Recently-determined crystal structures of SpyCEP, refined at only 2.8–3.1 Å resolution unveiled a modular protease that comprises nine distinct domains with the first five domains vital for catalytic activity [13]. Although a near full-length ectodomain SpyCEP construct was used for crystallization, several parts of the protein remained structurally uncharacterized, including the N-terminal region 34–114, the C-terminal region 1575–1613, and a more central region (214–272) containing the site of auto-cleavage.

Although a global priority, there is no licensed vaccine against *S. pyogenes*. Systematic proteomic analysis of *S. pyogenes* surface antigens identified SpyCEP as a leading vaccine candidate, conferring protection in a mouse challenge model after immunisation with a recombinant SpyCEP N-terminal fragment [14]. An immunoproteomic screen also identified SpyCEP as a leading target of an affinity-purified, protective anti-*S. pyogenes* human immunoglobulin preparation [15]. Vaccine-induced protection mediated by SpyCEP fragments has been observed in a various preclinical models, when used alone [16], [17], [18] or in combination with other antigens [19], [20], [21]. SpyCEP is surface-exposed, anchored to the bacterial cell wall and highly conserved between isolates [22], underlining its potential as a vaccine candidate. Vaccine-induced protection may, at least partially, reside in the ability of anti-SpyCEP antibodies to neutralise SpyCEP-mediated CXCL8 cleavage and reduce systemic dissemination of streptococci [23]. Binding of SpyCEP-specific IgG to the bacterial cell surface could also enhance complement-mediated phagocytic killing, further contributing to protection.

Detailed knowledge of antigen structure enables setup of appropriate in-process quality controls building confidence during vaccine manufacturing, and also provides an opportunity to fully employ structural vaccinology methods, by rationally redesigning antigens and epitopes to improve safety and efficacy [24], [25]. Despite the first glimpse into the SpyCEP structure [13], key questions regarding substrate interactions and dynamic properties remain. Although there is clear potential for SpyCEP to advance *S. pyogenes* vaccine development, structure-based antigen design has played a limited role in *S. pyogenes* research to date; most studies have used heavily truncated SpyCEP constructs [16], [17], [26], [27] or the wild-type recombinant protein with intact protease activity [19].

Inactivation of the SpyCEP protease activity by amino acid substitution of the residues constituting its catalytic triad should result in a safer single polypeptide vaccine antigen. However, an immature (proprotein) form of a catalytically-inactive SpyCEP may conceivably have altered structure and immunogenticity, and the ability of such a full-length mutated antigen to confer protection against *S. pyogenes* has not been reported previously. In the present study we set out to evaluate the ability of a full length SpyCEP protease-inactive mutant to confer vaccine-induced immunity, and to elucidate the structure and dynamics of this mutated vaccine antigen. We present the crystal structure of this novel inactive SpyCEP mutant and combine this with solution NMR and molecular dynamics data that shed further light on the mechanism of action of this chemokine protease and provide a framework for future structural vaccinology efforts.

## 2 Materials and methods

### 2.1 Expression and purification of SpyCEP N- and C-terminal domains

The SpyCEP_34-1613_ (D151A S617A) double mutant used in immunogenicity and crystallization experiments was previously cloned into pET-24b (Novagen) and purified from *E. coli* BL21(DE3) (New England Biolabs) soluble extracts as described [11]. SpyCEP N- (A34-Q244) and C-terminal (S245-A1613) domains were cloned into pET-28b (Novagen) utilising N- and C-terminal hexa-histidine tags, respectively. These constructs encompass the M1 strain SpyCEP ectodomain, excluding the leader peptide and LPXTG motif (Fig. S1). Two inactive mutants (D151A and S617A) were produced with the Q5 Site-Directed Mutagenesis Kit (New England Biolabs) and verified by DNA sequencing. [*U*-^15^N, ^13^C]-labelled SpyCEP_245-1613_ S617A or [*U*-^15^N]-labelled SpyCEP_34-244_ D151A and [*U*-^15^N]-labelled SpyCEP_245-1613_ S617A were produced by growing transformed *E. coli* BL21 (DE3) cells (New England Biolabs) in M9 enriched medium containing 50 μg/ml kanamycin and supplemented with ^15^NH_4_Cl (1 g/L) and ^12^C or ^13^C-glucose (2 g/L). The cultures were grown at 37 °C until an optical density at 600 nm of 0.8 was reached. Protein expression was induced by addition of 0.5 mM isopropyl β-d-thiogalactopyranoside (IPTG) and the cultures were grown at 18 °C overnight. The cells were harvested by centrifugation at 5000*g*, 4 °C for 15 min. [*U*-^15^N, ^13^C, ^2^D]-labelled SpyCEP_34-244_ D151A was expressed in Silantes OD2 CDN rich media under the same conditions.

The constructs were purified by resuspending cellular pellets in buffer A (50 mM Na phosphate pH 7.4, 500 mM NaCl, 20 mM imidazole) supplemented with a Complete, EDTA-free protease inhibitor tablet (Roche). The cells were disrupted by sonication and the lysate was clarified by centrifugation at 38,000*g*, 4 °C for 45 min. The clarified lysate was loaded onto a 5 ml HisTrap FF crude column (GE Healthcare) equilibrated with buffer A and eluted with a gradient of 20–500 mM imidazole. Fractions of interest were identified using SDS–PAGE, pooled, concentrated and recombined in a 1:1.5 M ratio (SpyCEP_245-1613_ S617A:SpyCEP_34-244_ D151A). Final separation was achieved with a Superdex 200 16/600 column (GE Healthcare) equilibrated with 20 mM MES pH 6.5, 150 mM NaCl. Adequate separation, observed by SDS–PAGE, was achieved. Homogeneity was assessed by native PAGE with the recombined sample migrating as one distinct band. N-terminal truncations (104–244, 109–244, 114–244, 116–244 and 121–244) and the C-terminal truncation (245–1578) were produced with the Q5 Site-Directed Mutagenesis Kit and verified by DNA sequencing. All mutants were expressed in the active form or with the D151A and S617A mutations.

### 2.2 Protein crystallization, structure determination, refinement and validation

Purified SpyCEP_34-1613_ (D151A S617A) protein was concentrated to 5 mg/ml and used for crystallization trials with a Crystal Gryphon liquid handling robot (Art Robbins Instruments) by mixing equal volumes (200 nl) of SpyCEP sample with crystallization reservoir solution. Crystals were obtained after roughly 2 weeks of incubation at room temperature (20 °C), in a condition containing 0.2 M ammonium formate and 20% (w/v) PEG 3350. These crystals were then mounted in cryo-loops, soaked in 20% ethyelene glycol (cryo-protectant) and stored in liquid nitrogen prior to X-ray data collection, which was performed at cryogenic temperatures (100 K) on beamline ID14-4 at the European Synchrotron Radiation Facility (ESRF), Grenoble, France. Data were processed using XDS [28] and other programs from the CCP4 [29] and PHENIX suites [30] revealing that crystals belonged to the monoclinic C2 space group and with cell parameters: a = 89.01 Å, b = 121.75 Å, c = 106.64 Å, and β = 111.78°. A Matthews coefficient of 2.38 Å^3^/Da, compatible with a solvent content of 48%, suggested the presence of one monomer in the asymmetric unit, as also confirmed by the molecular replacement (PHASER, [31]) solution obtained using coordinates from PDB 5XYR (crystal structure of SpyCEP from *Streptococcus pyogenes* strain JS95) as the search model. Manual model building and refinement were performed with Coot [32], BUSTER [33] and REFMAC5 [34] respectively, while the final model was inspected and validated using Molprobity [35]. Statistics for structure solution and refinement are reported in Table 1 (pdb:6VJB). Figures of structures were generated using the molecular graphic software Pymol (PyMOL Molecular Graphics System, version 2.1; Schrödinger, LLC; http://www.pymol.org).

**Table 1:** Data collection and refinement statistics.

Uniprot ID/Construct	Q9A180/SpyCEP D151A–S617A
PDB code	6VJB
Wavelength	0.93940
Resolution range (Å)*	89.01–2.24 (2.29–2.24)
Space group	*C* 1 2 1
Cell dimensions	
a, b, c (Å)	140.5, 121.75, 106.64
α, β, γ (°)	90, 111.78, 90
Total reflections	170,606 (7476)
Unique reflections	76,948 (3691)
Multiplicity	2.2 (2.0)
Completeness (%)	96.6 (78.6)
Mean I/sigma (I)	3.41 (1.6)
Wilson B-factor (Å^2^)	35.2
R_sym_	0.08 (0.56)
R_meas_	0.11 (0.79)
R_pim_	0.08 (0.56)
CC1/2	0.99 (0.56)
Reflections used in refinement	76,782 (6488)
Reflections used for R-free	3901 (326)
R_work_	0.193 (0.423)
R_free_	0.244 (0.515)
Number of non-hydrogen atoms	10,013
macromolecules	10,010
ligands	3
Protein residues	1316
RMS (bonds)	0.009
RMS (angles)	1.15
Ramachandran favored (%)	94.55
Ramachandran allowed (%)	5.45
Ramachandran outliers (%)	0
Rotamer outliers (%)	4.45
Clashscore	3.7
Average B-factor	52.0

*Highest resolution shell is shown in parenthesis.

R_sym_ = Σ_hkl_ Σ_i_|I_i_(hkl) − 〈I(hkl)〉|/Σ_hkl_ Σ_i_ I_i_(*hkl*).

R_work_ = Σ||F_(obs)_| − |F_(calc)_||/Σ|F_(obs)_|.

R_free_ = as for R_work_, but calculated for 5.0% of the total reflections that were chosen at random and omitted from refinement.

### 2.3 Assessment of SpyCEP immune responses in mice

Animal studies were conducted according to Italian Legislative Decree 116/1992 guidelines and approved by the internal Animal Welfare Body and the Italian Ministry of Health. Eight 5 week-old female CD1 mice were vaccinated intraperitoneally with 6 µg of SpyCEP_34-1613_ (D151A S617A) formulated in Alum. Sera collected two weeks after the second immunization and diluted 1:100, 1:4000 and 1:160000 in PBS containing 0.05% Tween 20 and 0.1% BSA were analyzed by ELISA for anti-SpyCEP total IgG content using SpyCEP_34-1613_ (D151A S617A) as plate coating antigen (at the concentration of 2 µg/ml in carbonate buffer). Pre-immune and post-2nd immunization sera were also tested in a CXCL8 cleavage ELISA assay [11] to evaluate their ability to block SpyCEP proteolytic activity. For *in vivo* protection experiments, groups of eight mice receiving three 20 µg vaccine doses were infected intranasally with GAS M1 strain 3348 on day 42 [19]. Mice were monitored on a daily basis for 1 week after treatment and euthanized when they exhibited defined humane endpoints that had been pre-established for the study in agreement with internal Animal Welfare Policies. Animals Fisher’s exact test was used for data statistical analysis.

### 2.4 NMR spectroscopy

All spectra were recorded on a Bruker Avance III HD 800 MHz or Bruker Avance III HD 950 equipped with triple-resonance cryoprobe. Spectral assignment of [*U*-^15^N, ^13^C, ^2^D]-labelled SpyCEP_34-244_ D151A complexed with unlabelled SpyCEP_245-1613_ S617A was performed at 60 μM in 20 mM MES pH 6.5, 150 mM NaCl, 10% D_2_O. The assignment covers 73 of the first 90 residues of the N-terminal domain. Spectral assignment of [*U*-^15^N, ^13^C]-labelled SpyCEP_245-1613_ S617A complexed with unlabelled SpyCEP_34-244_ D151A was performed at 50 μM in 20 mM MES pH 6.5, 150 mM NaCl, 10% D_2_O. The assignment covers the last 39 residues of the C-terminal domain. HN(CO)CACB [36] and HNCACB [37] triple resonance spectra were acquired at 283 K and at ^1^H frequency of 800 and 950 MHz.

Spectra were processed with NMRPipe [38] and analysed using CCPN Analysis version 2.4 [39]. Secondary chemical shifts were calculated with POTENCI [40], ncIDP [41] and the protocol of Kjaergaard et al. [42], [43]. Secondary structure propensity was calculated with ncSPC [41].

### 2.5 CXCL8 cleavage assay

Kinetic parameters of CXCL8 cleavage by SpyCEP were obtained using the Human IL-8/CXCL8 DuoSet ELISA kit (R&D Systems). Clear 96-well microplates (R&D Systems) were coated with capture antibody and incubated overnight at room temperature. Plates were washed three times with 400 µl 0.05% Tween in PBS using a Wellwash Microplate Washer (Thermo-Fisher). Wells were blocked with 300 µl 1% BSA in PBS for 2 h before being re-washed and loaded with 100 µl of prepared samples or CXCL8 standards for 2 h at room temperature. Standards were loaded in a 1:2 serial dilution from concentration 2000 pg/ml to 15.625 pg/ml. The wells were rewashed and incubated with detection antibody for 2 h at room temperature. The wells were rewashed and incubated with 100 µl Streptavidin–HRP for 20 min before being washed for a final time and incubated with 100 µl Substrate Solution. The reaction was stopped with the addition of 50 µl 1 M H_2_S0_4_ and optical density readings were obtained at 450 nm and 540 nm on a BMG FLUOstar Omega plate reader.

The assay was performed with SpyCEP_245-1613_ or SpyCEP_245-1578_ complexed with SpyCEP_34-244_ and Human CXCL8 was obtained from R&D Systems. Cleavage was examined using varying concentrations of CXCL8 (3–86 nM) incubated with 50 pM SpyCEP at 37 °C for 5 min and halted with 1 mg/ml Pefabloc SC plus (Sigma-Aldrich). Intact CXCL8 levels were measured using a CXCL8 standard curve, according to the manufacturer’s protocols. The rate of reaction was calculated as follows ([CXCL8]_t=0_ − [CXCL8]_t=5_)/time. The V_max_, k_cat_ and K_m_ were determined with Prism 7.05 (GraphPad). The negative control excluded SpyCEP and the positive control was a saturating concentration of CXCL8.

### 2.6 Molecular dynamics simulations

Atomistic simulations were run using the Gromacs 4.6.7 package [44] (www.gromacs.org) with the GROMOS53a6 force field [45]. The X-ray structure determined herein was used as the starting model, and crystallographic Ca^2+^ ions were retained. The system was solvated using the SPC water model, and ions were added to give a neutral system with a final NaCl concentration of 0.15 M with an initial box size of 13 × 13 × 13 nm. Periodic boundary conditions were applied, with a simulation time step of 2 fs. Equilibration runs were performed with duration of 1 ns and a time step of 2 fs. The protein backbone was restrained, pressure was coupled at 1 bar using the Berendsen barostat [46], and temperature was maintained at 310 K using a V-rescale thermostat [47] with a coupling constant of 0.1 ps. For the 100-ns production runs, the pressure was controlled at 1 bar through coupling to a Parrinello–Rahman barostat [48] with a coupling constant of 1 ps. Particle Mesh Ewald was used for long-range electrostatics [49] and the LINCS algorithm was used to constrain covalent bond length [50].

## 3 Results and discussion

### 3.1 Catalytically-inactive SpyCEP(D151A, S617A) double mutant elicits functional antibodies and confers protection in a mouse model of invasive disease

**Fig. 1:**
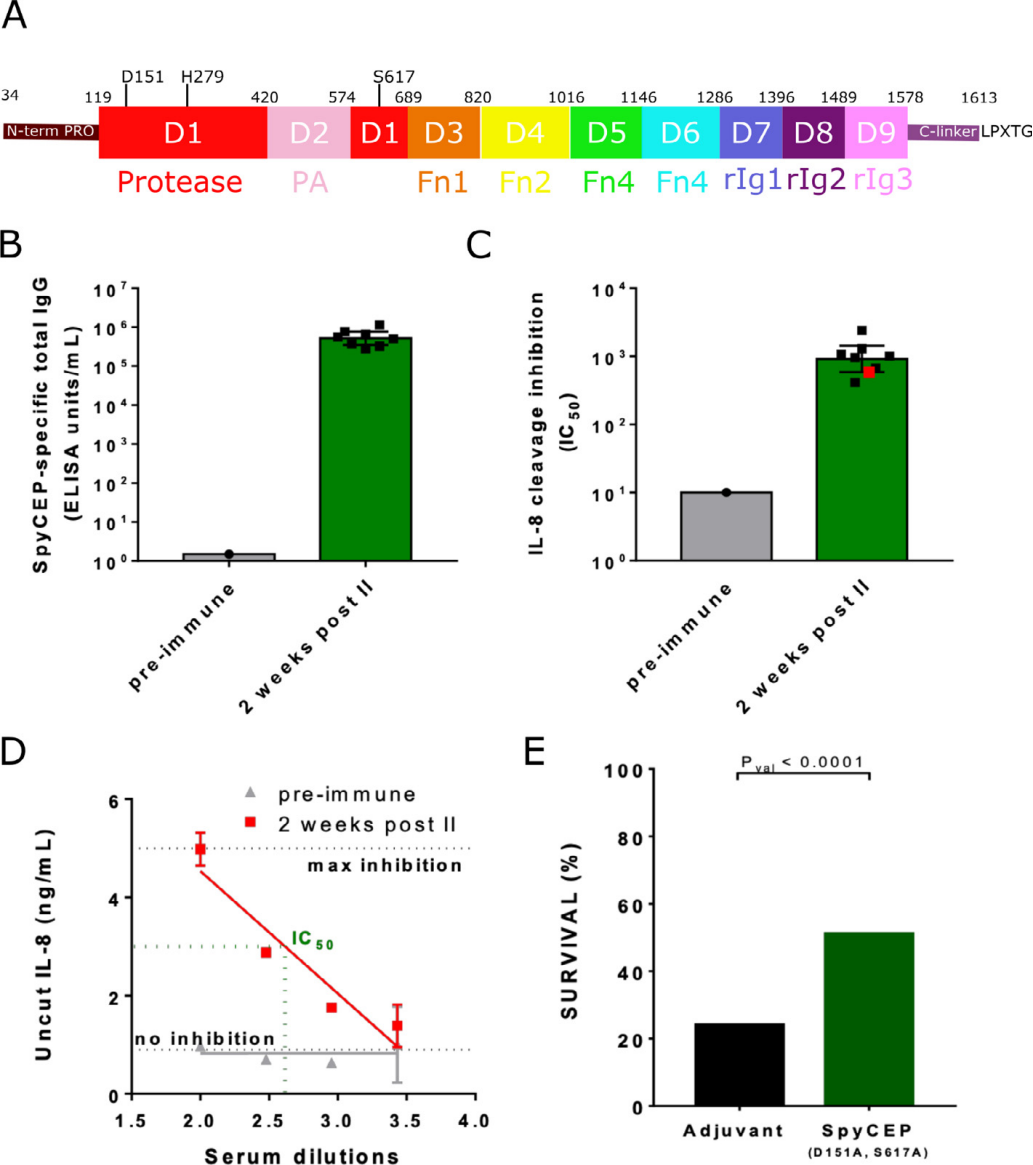
Immunogenicity and Protective activity of SpyCEP(D151A, S617A). (A) Domain architecture (B). Eight 5 weeks old female CD1 mice were vaccinated intraperitoneally with 200 µl of a formulation containing 6 µg of antigen and 2 mg/mL of Alum. Mice were immunized at study day 0 and 28. Approximately 100 µl bleeds (50 µl serum) were collected at day −1 (pooled sera). Final bleed was performed 2 weeks after the second immunization (sera maintained single) and mice were euthanized. Sera were analyzed by ELISA for anti-SpyCEP total IgG content. (C) Sera were tested in the IL-8/CXCL8 cleavage assay to evaluate their ability to block native SpyCEP proteolytic activity. The concentration of serum able to reduce of 50% the cleavage of CXCL8 is reported in the graph as IC50 value. (D) For pre immune serum and one selected post-2 serum (highlighted in red in panel C), the amount of uncut CXCL8 observed at each serum dilution tested is reported. (E) Five week old female CD1 mice were immunized intraperitoneally on days 0, 21 and 35 with 20 µg of SpyCEP (D151A, S617A) formulated in Alum or with Alum only as negative control. Mice were infected intranasally with GAS M1 strain 3348 on day 42 and survival was monitored for 6 days. Fisher’s exact test was used for statistical analysis. (For interpretation of the references to color in this figure legend, the reader is referred to the web version of this article.)

Since an active protease may be considered a safety risk as a human vaccine antigen, we evaluated the potential of a genetically-engineered inactivated form of full-length SpyCEP to act as a vaccine candidate (Fig. 1A). CD1 mice immunized with the SpyCEP_34-1613_ (D151A, S617A) double mutant 170 kDa polypeptide mounted a rapid serological response to the antigen (Fig. 1B). We chose this double mutant of the catalytic triad as previous work had demonstrated that these mutations had minimal effect on structure while rendering the enzyme not only catalytically inactive but able to form a fully folded enzyme [11]. The immune sera provided dose-dependent inhibition of CXCL8 cleavage by wild-type SpyCEP (Fig. 1C, D). The geometric mean concentration of sera able to reduce 50% of CXCL8 cleavage (IC_50_) was 2 logs higher after two vaccine doses compared to pre-immune sera (Fig. 1C, D). The capacity of the SpyCEP_34-1613_ (D151A, S617A) double mutant to mediate protection against M1T1 GAS intranasal challenge was evaluated in mice receiving three vaccine doses over 42 day, or adjuvant alone as control. Significant levels of protection were achieved for the vaccinated group compared to the negative control (Fig. 1E). Together, these data show that the catalytically-inactive SpyCEP double mutant can confer protection similar to that reported previously [16], [17], [18], and that the mechanism of vaccine-induced protection at least partially involves inhibition of the natural SpyCEP-mediated chemokine inactivation process. These findings suggest that the SpyCEP double-mutant is a valid candidate for human vaccine development.

### 3.2 Crystal structure of SpyCEP(D151A, S617A)

We next completed the structure determination of the full length SpyCEP vaccine construct. Extensive screening using crystals suitable for X-ray diffraction ultimately yielded a novel structure of the full-length ectodomain harbouring the double mutation in the active site, SpyCEP(D151A, S617A) [11], at 2.2 Å resolution, a notable improvement on the resolution of the three previously reported structures [13]. A series of additional SpyCEP constructs were expressed in and purified from *Escherichia coli* and subjected to crystallization screens; N- and C-terminal truncations were created based on the related C5a peptidase structure (excluding residues 1132 onwards) and some predicted disorder within residues 33–112 [51], [52]. Attempts to optimise microcrystals for the truncated constructs failed to yield improved crystals, suggesting that the regions removed contain important features for structure or stability, or mediate crystal contacts. A complete diffraction data set to 3.3 Å resolution was collected previously [53], however past attempts to solve the structure using molecular replacement with existing structures of related proteases or by single-wavelength anomalous dispersion (SAD) failed [53]. Further crystal optimization and the availability of a recent X-ray structure of wild-type SpyCEP and of a different catalytically inactive mutant (H279A, S617A) [13] allowed us find a positive molecular replacement solution for our SpyCEP diffraction data. The X-ray data collection and refinement statistics are summarized in Table 1.

**Fig. 2:**
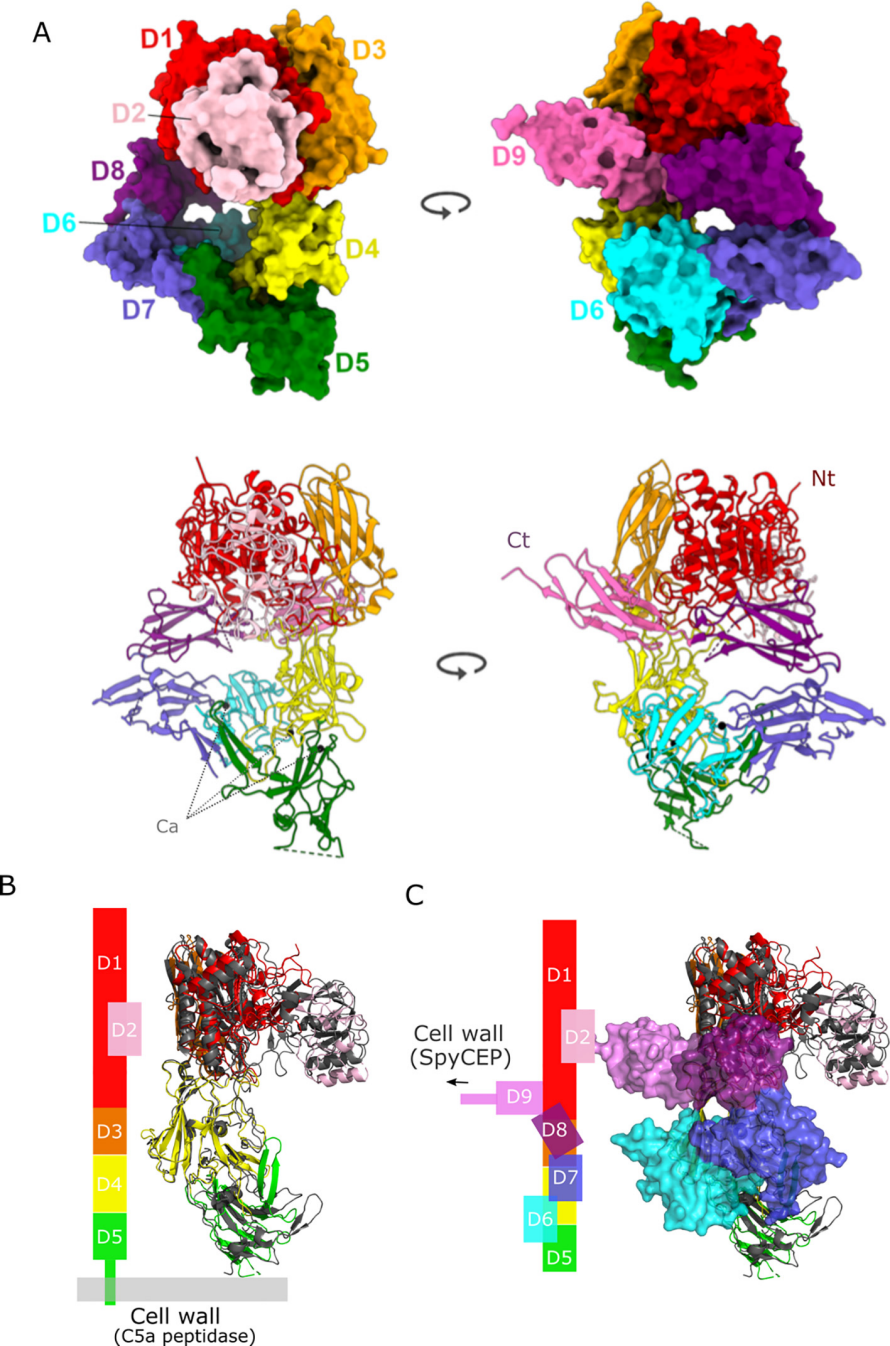
Structure of SpyCEP(D151A, S617A). (A) Cartoon representation of the structure of SpyCEP(D151A, S617A) in two orthogonal orientations. Domains are coloured according to Fig. 1A and termini are indicated. (B) Cartoon representation of the superposition of the structure of C5a peptidase (pdb:1xf1 or 3eif; colored in grey) on the the first 5 domains of SpyCEP(D151A, S617A). Schematic representation of the relative position of the domains and the cell wall anchor isshown on the left. (C) Superposition of the structure of C5a peptidase (colored in grey) on SpyCEP(D151AS617A) with domains D5–D9 shown in surface representation. Schematic representation of the relative position of the domains and the cell wall anchor is shown on the left.

The domain architecture of the SpyCEP(D151A, S617A) mutant matches that observed in the published SpyCEP structures [13], as expected, with nine distinct folded domains (D1–D9; Fig. 2). A search of the protein data bank (PDB) using the DALI program [54] revealed that the overall structure is most similar to that of wild-type SpyCEP (pdb:5XYR), with a root mean square deviation (RMSD) of 1.4 Å, obtained by superposing with 1248 equivalent C_α_ atoms. The entire protease domain (D1) encompasses L119-G689 and is located at the mature N terminus. It is interrupted by an insertion domain (D2) between K420-N574 that is termed the protease-associated (PA) domain. The core protease domain belongs to the canonical subtilisin family with RMSDs in the range of 1.9–2.0 Å over available subtilisin Carlsberg structures.

The next three domains of SpyCEP (D3:L690-F820, D4:E821-D1016 and D5:R1017-E1146) are fibronectin-like domains, comprising a core β-sandwich with a Fibronectin Type III fold. An extensive interdomain interface locks the relative position of the protease and the first of the fibronectin domains (Fn1). The inter β-strand regions of D4 and D5 contain elaborate loops and secondary structure elements, which facilitate interdomain contacts and maintain their relative orientations. This whole element (D1–D5) appears to be rigid as the same arrangement of the first three fibronectin domains is also present in the C5a peptidases from *S. pyogenes* and *S. agalactiae* [51], [52] (RMSD 3.2 Å over 799 C_α_ atoms), and in this context could provide a rigid stalk to project the protease machinery (~100 Å) from the C-terminal cell wall anchor (Fig. 2C), presumably enhancing its functionality.

SpyCEP is significantly larger than the C5a peptidase and has an additional four domains (D6:T1147-K1286; D7:K1287-K1396; D8:D1397-M1489; D9:L1490-A1578) before the linkage to the cell wall. D6 also belongs to the Fn Type III family, whereas the final three domains (D7–D9) are similar to immunoglobin-like (Ig) folds, but adopt the reversed strand order and are therefore termed reversed Ig-like domains. These are commonly seen in sortase-assembled pilin domains from Gram positive bacteria [55], where they perform a role in host discrimination and cell adhesion.

Surprisingly, the two C-terminal domains (D8 and D9) in SpyCEP coil back on the structure and contact the protease domain (Fig. 2B), an interaction that might retain this distal protease domain proximal to the cell wall. Furthermore, three tightly bound calcium ions present within the interfacial regions of D4–D7 (Fig. 2A) also appear to stabilise the overall architecture of SpyCEP. Calcium regulation is a feature often seen in the related cell envelope proteases of lactic acid bacteria, for example PrtS in *S. thermophilus* [56]. It is therefore conceivable that host environmental and signalling events that result in changes in local calcium concentrations, may facilitate a conformational switch from condensed coiled arrangement of D4–D9 to a more extended one, thereby facilitating enhanced presentation of the protease domain, akin to the unbending of integrins [57].

### 3.3 SpyCEP contains N- and C-terminal intrinsically disordered regions

The functional SpyCEP heterodimer is considered to span residues 34–1613, excluding the signal peptide (residues 1–33) and the LPXTG cell wall-anchoring motif (residues 1614-1647) [13], [53]. However, electron density was only observed for residues 116–1574 of the ectodomain (MW ~160 kDa) in all SpyCEP crystal structures, with residues 34–115 and 1575–1613 remaining uncharacterized. IUPRED analysis predicts that these missing regions are disordered [58]. Moreover, a recombinant N-terminal fragment of SpyCEP spanning residues 31–245 was previously studied by CD spectroscopy and indicated the presence of a random coil denatured protein [11]. To shed light on these observations experimentally, we used solution NMR to explore these terminal regions and assess their structural propensity, dynamics and expose undetermined functional roles.

**Fig. 3:**
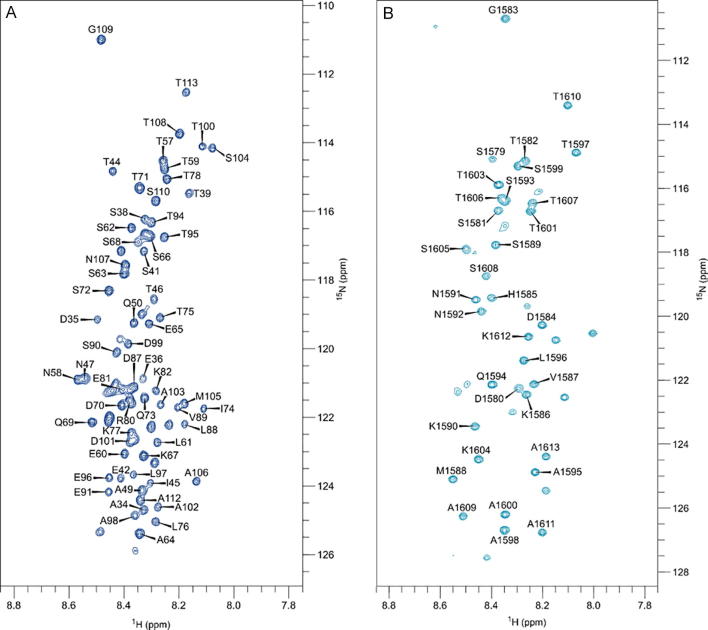
Assigned NMR spectra of SpyCEP. ^1^H, ^15^N-HSQC spectra and assignment of (A) [*U*-^15^N]-labelled SpyCEP_34-244_ D151A complexed with unlabelled SpyCEP_245-1613_ S617A, (B) [*U*-^15^N]-labelled SpyCEP_245-1613_ S617A complexed with unlabelled SpyCEP_34-244_ D151A. The N-terminal assignment (left) covers residues 34–113 of the SpyCEP ectodomain. The C-terminal assignment (right) covers residues 1578–1613 of the SpyCEP ectodomain.

The ability to produce a heterodimeric version of catalytically inactive SpyCEP provides an opportunity to study each domain independently while in complex with an unlabelled complementary domain. Furthermore, any highly flexible regions should be observable with standard heteronuclear NMR methodology despite the large overall molecular weight. The core structured unit of SpyCEP with a molecular mass of >160 kDa would not be observable in non-TROSY NMR approaches due to its slow rotational tumbling in solution.

(^1^H, ^15^N) HSQC spectra of uniformly [*U*-^15^N]-labelled SpyCEP_34-244_ D151A and [*U*-^15^N]-labelled SpyCEP_245-1613_ S617A in complex with their complementary unlabelled subunits exhibited narrow proton chemical shift dispersion, which indicated the presence of significant disorder within these regions. Also, the fact that these regions were observed in NMR spectra indicated extensive dynamics on a pico- to nano-second timescale [59] (Fig. 3). Notwithstanding the large size of the SpyCEP heterodimer, 81 and 45 peaks were observed in spectra of the N- and C-terminal subunits, respectively. A triple-resonance backbone resonance assignment of the ^13^C^α^, ^13^C^β^, ^1^H_N_ and ^15^N nuclei was performed at 283 K to determine the sequence context of the disorder (Fig. 3). The N- and C-terminal assignments map to residues spanning 34–113 and 1578–1613 of the SpyCEP ectodomain, respectively. These assignments highlight the high degree of conformational flexibility experienced by both regions within the context of full-length SpyCEP, and likely explain the absence of electron density for these regions in any of the crystal structures determined to date.

**Fig. 4:**
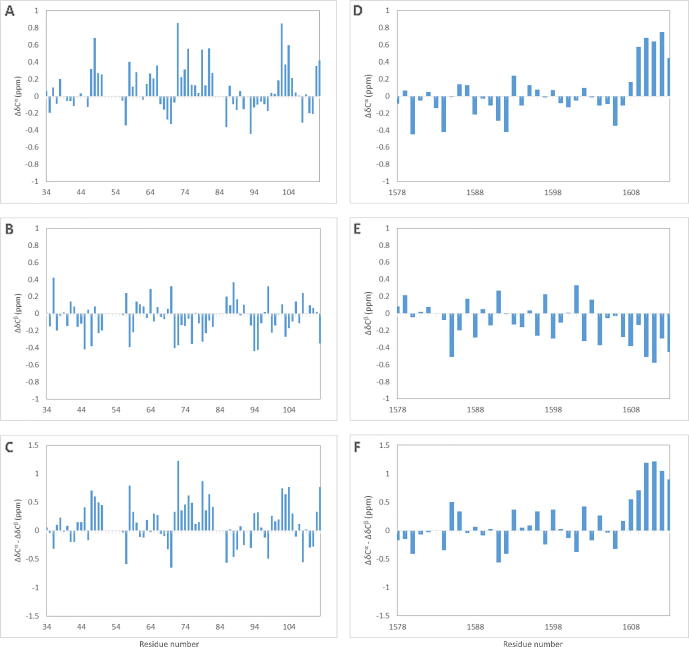
Chemical shift indicators of secondary structure in SpyCEP. ^13^C secondary chemical shifts of the SpyCEP N- and C-terminal disordered regions. Secondary chemical shifts are shown for (A) ^13^C^α^ (B) ^13^C^β^ and (C) ^13^C^α^–^13^C^β^ secondary chemical shifts calculated for the N-terminus, and exhibit helical propensity in residues 47–50, 71–82 and 99–107. (D) ^13^C^α^ (E) ^13^C^β^ and (F) ^13^C^α^–^13^C^β^ secondary chemical shifts calculated for the C-terminal linker exhibit helical propensity only from residue 1608.

Secondary chemical shifts, the difference between experimentally derived chemical shifts and residue-specific random coil chemical shifts, are an invaluable tool to detect transient and short-ranged secondary structure within intrinsically disordered proteins. Secondary chemical shifts derived from ^13^C backbone nuclei are sensitive reporters of secondary structure, with ^13^C^α^ and ^13^C′ reporting α-helical (positive values) or β-strand (negative values) propensity, while ^13^C^β^ chemical shifts exhibit the inverse relationship [60]. ^13^C^α^ and ^13^C^β^ secondary chemical shifts were calculated for SpyCEP resonance assignments [42], [43] (Fig. 4). Residues 47–50, 71–82 and 99–107 exhibited pronounced ^13^C^α^ secondary shifts and negative ^13^C^β^ shifts, indicating a persistent helicity in the N-terminal disordered region. Secondary chemical shifts calculated for the C-terminal disordered region are featureless until residue 1608, where a helical propensity is observed in 6 residues immediately prior to the site of membrane association (Fig. S2). These data indicate that the N-terminal intrinsically disordered region (IDR) displays significant helical propensity, which is likely to be important for the secretion of active SpyCEP and to guide the folding process. In contrast, the C-terminal IDR exhibits little secondary structure propensity and is therefore completely unstructured except at the very C-terminus, which is recognised by the sortase anchoring machinery.

N- and C-terminal domain truncations were designed to assess the role of disorder on the SpyCEP_34-1613_ heterodimer, utilising backbone assignment data and sequence alignment with close relatives from other *Streptococcus* species. N-terminal truncations were unstable and could not effectively complex with SpyCEP_245-1613_. A C-terminal truncation, SpyCEP_245-1578_, expressed at a higher level than SpyCEP_245-1613_ and was reconstituted with SpyCEP_34-244_ to generate a stable dimer. The catalytic activity of this new construct SpyCEP_34__-__1578_ was compared with SpyCEP_34-1613_ to determine the relevance of C-terminal disorder to enzyme activity. SpyCEP mediated CXCL8 degradation was assessed with an enzyme-linked immunosorbent assay (ELISA) (Fig. S3). K_M_ values were consistent between the two SpyCEP versions, suggesting C-terminal disorder beyond D9 has little impact on CXCL8 binding or catalytic activity. However, SpyCEP can be released from the bacterial surface as a soluble enzyme and it is likely that flexibility in the C-terminal linker plays a role in facilitating this event [6], [8].

The bacterial cell wall is peppered with proteins and peptidoglycan that extend from the surface. The disordered linker upstream of the LPXTG cell wall-anchoring motif offers greater degrees of conformational freedom and distance from site of attachment than a structured region of the same size. If extended, this region could stretch to beyond the cell envelope dimension of 40–60 nm, however any compaction or additional cell wall interactions may restrict the accessibility of the protease domain. Thus, the ability to further extend the protease head away from the bacterial surface, with a host-induced unwrapping of the tandem domains could provide an effective mechanism for *in situ* regulation of protease activity.

### 3.4 Dynamics within the PA domain may provide interdomain communication

**Fig. 5:**
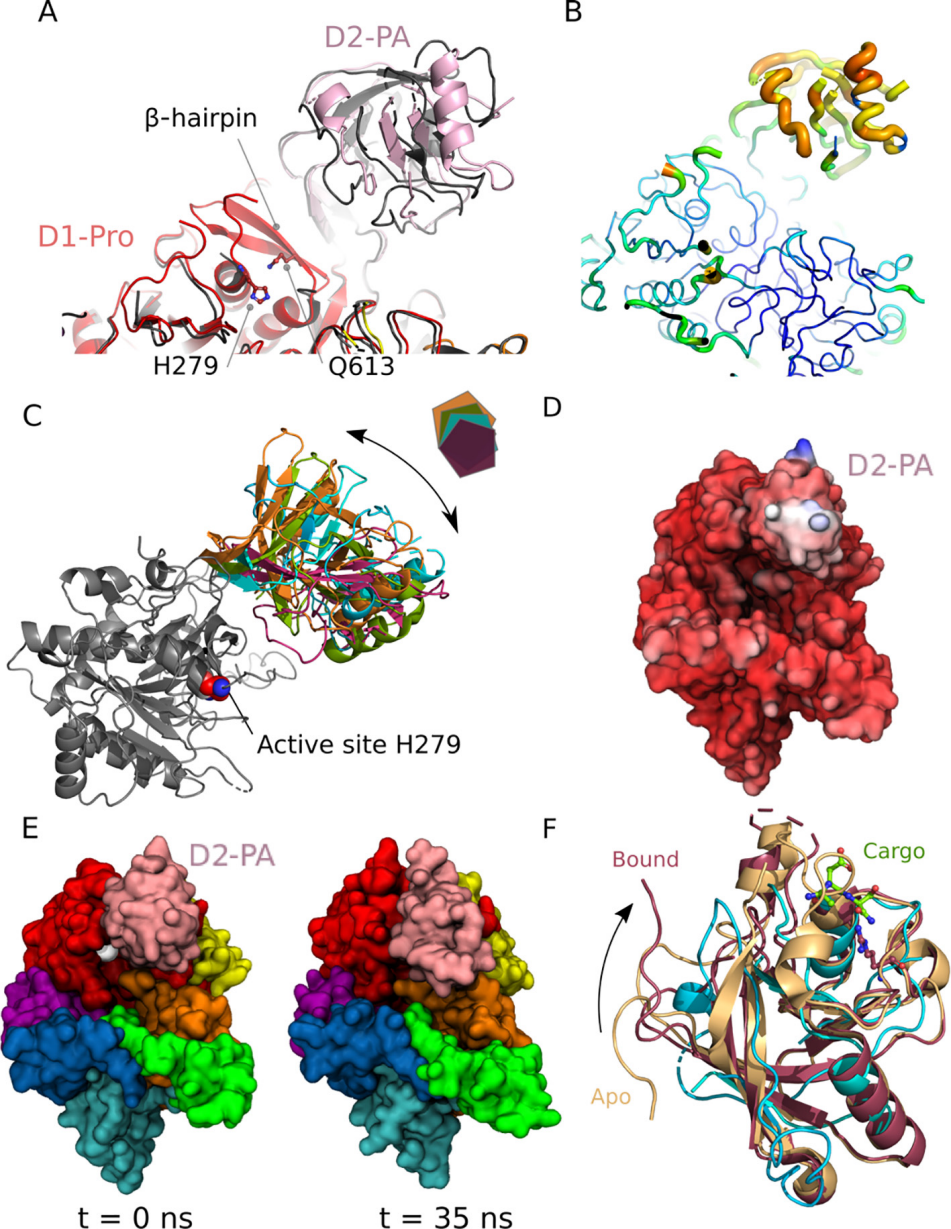
PA domains are conformationally adaptable. (A) Pairwise superposition of the structure of the protease and PA domains from SpyCEP(D151A, S617A), with equivalent region from the published SpyCEP(H279A, S617A) structure (pdb: 5XXZ). (B) Putty representation of SpyCEP(D151A, S617A) D1 and D2 with increasing B-factors indicated by the diameter of the worm and its color (blue to red). (C) Superposition of the relative orientation of the Protease and PA domains from SpyCEP(D151A, S617A) (cyan) with representative related subtilase structures C5a, (pdb: 1xf1 and 3eif, orange and green) and curcumisin (pdb: 3vta, purple). (D) Simulations of SpyCEP(D151A, S617A) indicate mobility of PA domain, protein coloured according to RMSD over the simulation (blue largest, red lowest). (E) The active site (H279 shown in white) is solvent accessible in starting model (t = 0 ns) however motion of the PA domain coupled with loop motions leads to closure of the active site entrance (snapshot at 35 ns shown). Domains coloured according to Fig. 1. (F) Superposition of PA domain from SpyCEP(D151A, S617A) (cyan) with the PA domain from the vacuole sorting receptor alone (pdb: 4tjv, gold) and bound (pdb: 4tjx, dark red) to peptide cargo (green sticks). (For interpretation of the references to color in this figure legend, the reader is referred to the web version of this article.)

Protease-associated (PA) domains are rather enigmatic. First identified in the subtilisin family of proteases, the finer details of their roles are unclear, although evidence exists to suggest the PA domain provides additional substrate specificity outside the catalytic site [61], [62], [63], [64]. However, no structures exist for a protease PA-substrate complex. In the pyrolysin-like subtilases from tomato plants the PA domain was shown to perform a role in homo-dimerization and regulation [63]. While no evidence exists for dimerization of SpyCEP, the insertion of the PA domain directly above the catalytic site suggests a role in regulating substrate access (Fig. 5). An analysis of our structure and other key PA-containing proteases highlights some mobility within the PA domain that might be important for regulating protease activity.

Surprisingly, the structure of the double mutant described here, SpyCEP(D151AS617A), is most similar to the wild-type (PBD: 5XYR) and not to the published structure for the alternative double mutant (H279A, S617A; PBD: 5XXZ). Significant structural differences exist between the two double mutants, and these localise to the PA domain and a neighbouring ß-hairpin that supports the relative positions of the PA and protease domains (Fig. 5A). This region is completely disordered in SpyCEP (H279A, S617A) as native contacts between the catalytic H279 and Q613 at the base of the ß-hairpin are removed by mutation of the histidine to alanine (Fig. 5A). The differences in the PA domain conformation are likely driven by the loss in structure of this supporting hairpin, which highlights a potential allosteric link to the catalytic site. Such allostery suggests interdomain communication may play a role in enzyme activation.

Strikingly, in solved structures of proteases with PA domains, the PA domains generally display higher B-factors than the main protease structure, suggesting a degree of intrinsic flexibility for this region. Furthermore, in related structures of subtilases the relative orientation of PA domains with respect to the main body of the protease varies significantly (Fig. 5B and C). In light of this, refinement of our SpyCEP structure by using the PA domain and the rest of the protein as separate search models for molecular replacement gave significant improvements in electron density maps over using the whole SpyCEP structure, and a shift in its relative position was observed compared to the published structures. Taken together, these observations suggest that PA domains are conformationally mobile, and that this feature is likely to be important for function. To further investigate the role of PA conformational dynamics we performed molecular dynamics simulations of SpyCEP (D151A, S617A) and supplemented this with normal mode analysis (NMA) using the elNémo server to probe low frequency motions [65]. In molecular dynamics the greatest mobility is observed for the PA domain (Figs. 5D and S4). Inspection of the residues surrounding the catalytic H279 indicate that motion of the PA domain would modulate access to the active site (Fig. 5E). B-factors computed from the mean square atomic displacements for the slowest 100 normal modes show significantly elevated values for the PA domain (Fig. S5). This pattern matches those observed in the experimental B-factors for SpyCEP and other PA-domain protease structures and suggests mobility is an inherent feature of PA domain function.

As PA domain peaks are absent from ^1^H-^15^N HSQC NMR spectra of SpyCEP (Fig. 3), PA domain mobility is unlikely to be dynamic on a fast (sub-microsecond) timescale, unlike motions in the termini. Instead, it is likely that the PA domain adopts distinct stable conformations that can interconvert, perhaps driven by binding to an interaction partner. This notion is reminiscent of other, unrelated PA-containing families of proteins, for example the vacuole trafficking receptors and E3 ubiquitin ligases [66], [67], where the domains play a role in cargo recruitment, delivery and presentation. In the only available structure of a PA domain complex, the PA domain of the vacuole sorting receptor (VSR) from *Arabidopsis thaliana* (VSR1PA) undergoes a large rearrangement of its N- and C-terminal regions upon binding cargo peptide [66]. Cargo binding induces local conformational changes that propagate allosterically from the cargo binding loop to the C terminus via a network of residues in switch (I-IV) loop regions (Fig. 5F). Intriguingly, a crucial R95 residue that makes numerous contacts with the N terminus of the cargo peptide is conserved across the wider PA family, including the bacterial subtilases (Fig. S6) [64]. It is therefore conceivable that the PA domain of SpyCEP recognises the N-terminal ELR motif in CXC substrates *via* a similar binding mode and a subsequent conformation change delivers the C-terminal cleavage site to the catalytic triad.

## 4 Concluding remarks

Structural and functional understanding of the SpyCEP protein have enabled rational design of a catalytically-inactive mutant which was herein demonstrated to be an effective protective immunogen in a mouse challenge model of GAS infection. Extensive structural analyses provided several insights into SpyCEP biological function and have improved the level of characterization of this vaccine antigen. In particular, crystal structures of SpyCEP are strongly indicative of a functional mechanism that involves a significant conformational change to modulate the accessibility of the active site. We propose a model in which the PA domain provides the initial point for CXCL8 docking, which induces a subsequent conformational change for optimal delivery of the C terminus to an opened active site. Our data facilitate and lend confidence to the development of a protective GAS vaccine using the existing double mutant SpyCEP(D151A, S617A) antigen. Moreover, the new molecular details provide the basis for both a deeper structure-guided approach to design of next-generation SpyCEP antigens and to the rational design of small-molecule active site therapeutic protease inhibitors to treat GAS diseases.

## CRediT authorship contribution statement

**Sophie McKenna:** Investigation, Formal analysis, Data curation, Writing - review & editing, Visualization. **Enrico Malito:** Investigation, Formal analysis, Data curation, Writing - review & editing, Visualization. **Sarah L. Rouse:** Investigation, Formal analysis, Data curation, Writing - review & editing, Visualization. **Francesca Abate:** Writing - review & editing, Supervision. **Giuliano Bensi:** Supervision, Writing - review & editing. **Emiliano Chiarot:** Supervision, Writing - review & editing. **Francesca Micoli:** Supervision, Writing - review & editing. **Francesca Mancini:** Supervision, Writing - review & editing. **Danilo Gomes Moriel:** Supervision, Writing - review & editing. **Guido Grandi:** Supervision, Writing - review & editing. **Danuta Mossakowska:** Supervision, Writing - review & editing, Project administration. **Max Pearson:** Investigation, Formal analysis, Data curation, Writing - review & editing, Visualization. **Yingqi Xu:** Investigation, Formal analysis, Data curation, Writing - review & editing, Visualization. **James Pease:** Supervision, Writing - review & editing, Project administration. **Shiranee Sriskandan:** Supervision, Writing - review & editing, Project administration. **Immaculada Margarit:** Investigation, Formal analysis, Data curation, Writing - review & editing, Visualization. **Matthew J. Bottomley:** Conceptualization, Methodology, Writing - original draft, Project administration. **Stephen Matthews:** Conceptualization, Methodology, Writing - original draft, Project administration.
