# Supplementary material for: Structure, dynamics and immunogenicity of a catalytically inactive CXC chemokine-degrading protease SpyCEP from Streptococcus pyogenes
Source: Comput Struct Biotechnol J. 2020 Mar 13;18:650–60. doi: 10.1016/j.csbj.2020.03.004 (PMC7113628; doi:10.1016/j.csbj.2020.03.004)
Supplement: Supplementary data 2 [file mmc2.docx]

**Supplementary information**

ADELSTMSEPTITNHAQQQAQHLTNTELSSAESKSQDTSQITLKTNREKEQSQDLVSEPTTTELADTDAASMANTGSDATQKSASLPPVNTDVHDWVKTKGAWDKGYKGQGKVVAVIATGIDPAHQSMRISDVSTAKVKSKEDMLARQKAAGINYGSWINDKVVFAHNYVENSDNIKENQFEDFDEDWENFEFDAEAEPKAIKKHKIYRPQSTQAPKETVIKTEETDGSHDIDWTQTDDDTKYESHGMHVTGIVAGNSKEAAATGERFLGIAPEAQVMFMRVFANDIMGSAESLFIKAIEDAVALGADVINLSLGTANGAQLSGSKPLMEAIEKAKKAGVSVVVAAGNERVYGSDHDDPLATNPDYGLVGSPSTGRTPTSVAAINSKWVIQRLMTVKELENRADLNHGKAIYSESVDFKDIKDSLGYDKSHQFAYVKESTDAGYNAQDVKGKIALIERDPNKTYDEMIALAKKHGALGVLIFNNKPGQSNRSMRLTANGMGIPSAFISHEFGKAMSQLNGNGTGSLEFDSVVSKAPSQKGNEMNHFSNWGLTSDGYLKPDITAPGGDIYSTYNDNHYGSQTGTAMASPQIAGASLLVKQYLEKTQPNLPKEKIADIVKNLLMSNAQIHVNPETKTTTSPRQQGAGLLNIDGAVTSGLYVTGKDNYGSISLGNITDTMTFDVTVHNLSNKDKTLRYDTELLTDHVDPQKGRFTLTSHSLKTYQGGEVTVPANGKVTVRVTMDVSQFTKELTKQMPNGYYLEGFVRFRDSQDDQLNRVNIPFVGFKGQFENLAVAEESIYRLKSQGKTGFYFDESGPKDDIYVGKHFTGLVTLGSETNVSTKTISDNGLHTLGTFKNADGKFILEKNAQGNPVLAISPNGDNNQNFAAFKGVFLRKYQGLKASVYHASDKEHKNPLWVSPESFKGDKNFNSDIRFAKSTTLLGTAFSGKSLTGAELPDGHYHYVVSYYPDVVGAKRQEMTFDMILDRQKPVLSQATFDPETNRFKPEPLKDRGLAGVRKDSAFYLERKDNKPYTVTINDSYKYVSVEDNKTFVERQADGSFILPLDKAKLGDFYYMVEDFAGNVAIAKLGDHLPQTLGKTPIKLKLTDGNYQTKETLKDNLEMTQSDTGLVTNQAQLAVVHRNQPQSQLTKMNQDFFISPNEDGNKDFVAFKGLKNNVYNDLTVNVYAKDDHQKQTPIWSSQAGASVSAIESTAWYGITARGSKVMPGDYQYVVTYRDEHGKEHQKQYTISVNDKKPMITQGRFDTINGVDHFTPDKTKALGSSGIVREEVFYLAKKNGRKFDVTEGKDGITVSDNKVYIPKNPDGSYTISKRDGVTLSDYYYLVEDRAGNVSFATLRDLKAVGKDKAVVNFGLDLPVPEDKQIVNFTYLVRDADGKPIENLEYYNNSGNSLILPYGKYTVELLTYDTNAAKLESDKIVSFTLSADNNFQQVTFKITMLATSQITAHFDHLLPEGSRVSLKTAQDQLIPLEQSLYVPKAYGKTVQEGTYEVVVSLPKGYRIEGNTKVNTLPNEVHELSLRLVKVGDASDSTGDHKVMSKNNSQALTASATPTKSTTSATAKA

**Figure S1:** SpyCEP_34-1613_ ectodomain used in NMR studies. The full sequence is shown here with D151A and S617A mutations in red and the autocatalytic processing site in green. The N- and C-terminal constructs included N- and C-terminal hexa-histidine tags, respectively but numbering of the SpyCEP sequence was in line with ectodomain numbering to simplify discussion. The underlined sequences display assignment coverage in both domains.


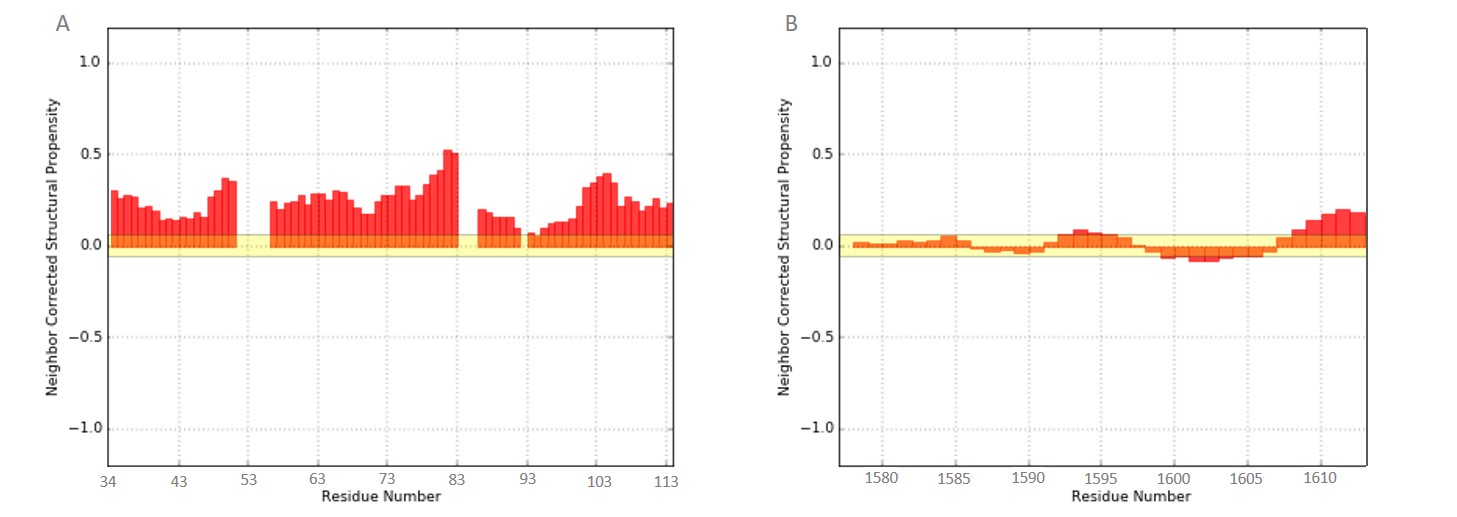


**Figure S2:** ncSPC structural propensity prediction plots of the N- and C-terminal IDRs of SpyCEP. A score between +1 and -1 indicates a propensity to form 100% α-helical or 100% β-strand, respectively. (A) The structural propensity in the N-terminal IDR deviates from the predicted random coil state (yellow band), indicating helical propensity along the sequence. (B) The C-terminal IDR is largely devoid of any local secondary structure as the structural propensity adheres closely to the predicted random coil state. Slight helical propensity (< 25%) is observed from residue 1608.

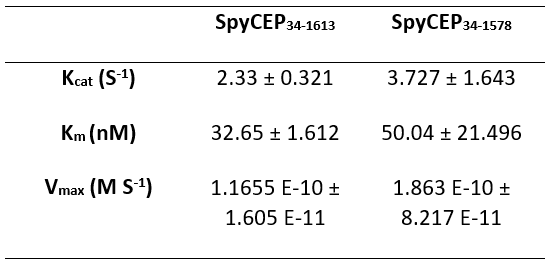


**Figure S3:** Michaelis-Menten plots of (**A**) SpyCEP_34-1613_ and (**B**) SpyCEP_34-1578_ mediated degradation of CXCL8. The substrate concentration in nM is plotted against the velocity in M s^-1^. Experiments were run in duplicate and data shown separately as circles and triangles. N=2 experimental replicates per data point and error bars represent SD. The mean values for kinetic parameters V_max_, k_cat_ and K_m_ ± SD are reported in the table

**
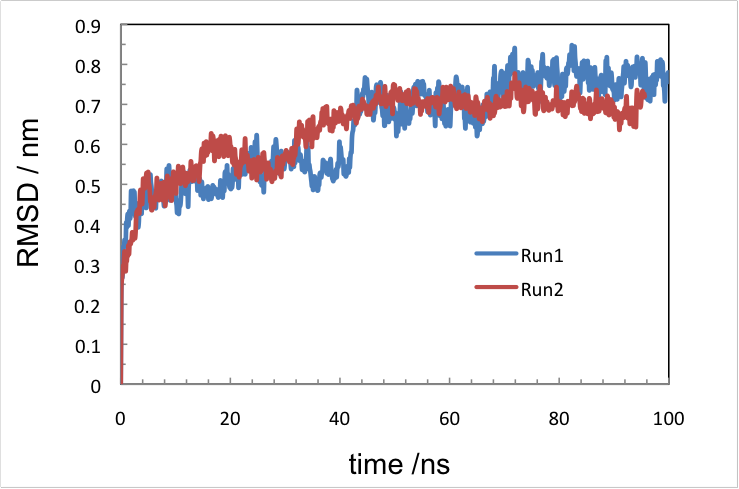
**

**Figure S4:** Time evolution of RMSD values for Cα atoms with respect to starting structure during MD simulations. Equilibrium has been established for both runs after 70ns.


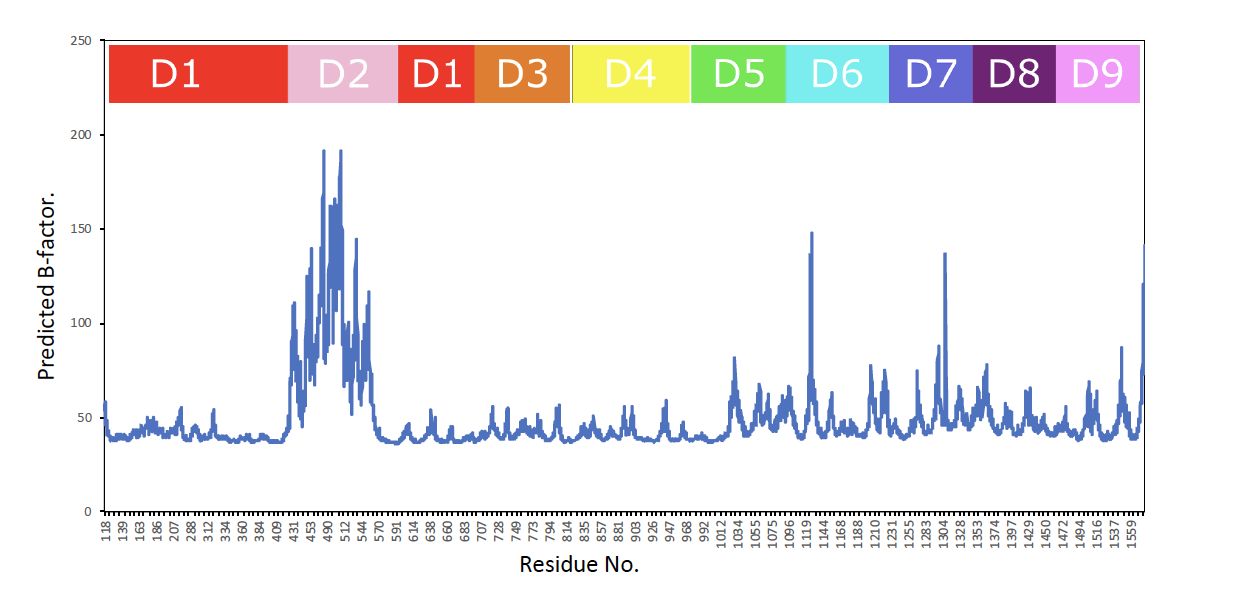


**Figure S5:** B-factors (Å^2^) calculated mean square atomic displacement of the first 100 lowest-frequency normal modes and plotted against residues number. Elevated values for D2 (the PA domain) suggest inherent domain molility.


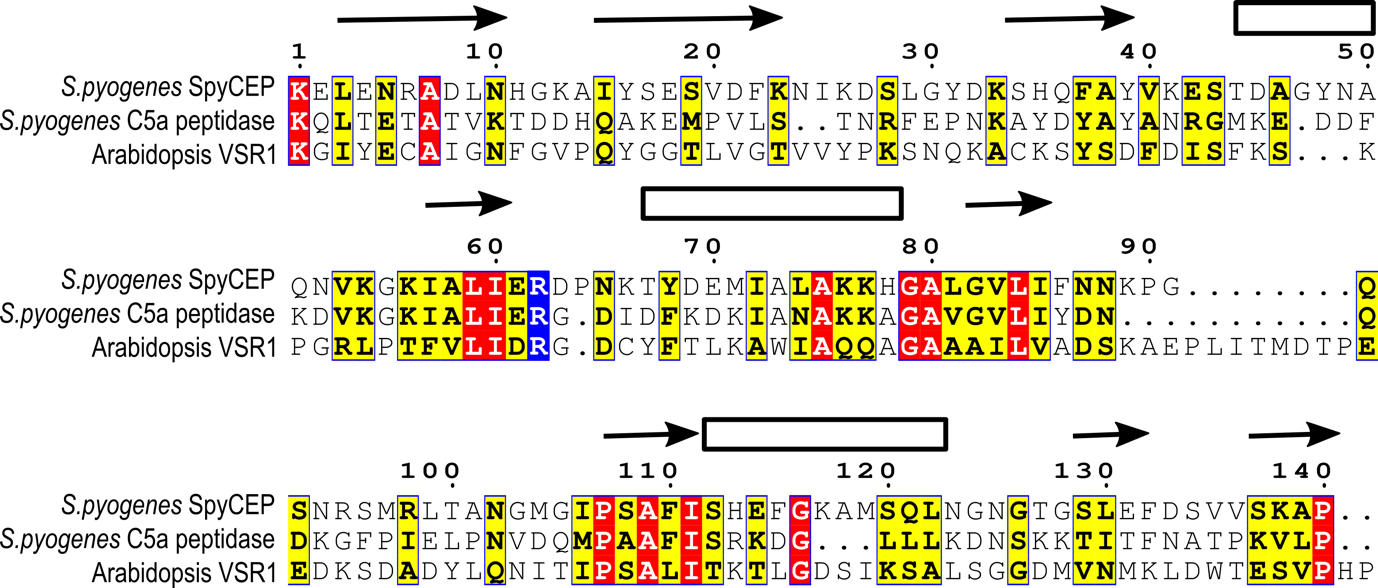


**Figure S5: Sequence alignment for key PA domains.** The conserved arginine that plays an essential role in the conformational switch in the Vacuolar Sorting Receptor (VCR1) is shaded blue. Total conservation is otherwise shaded red and these largely coincide with secondary structure elements.
